# Development and validation of a predictive model for post-endoscopic retrograde cholangiopancreatography cholangitis: A risk factors based nomogram

**DOI:** 10.1097/MD.0000000000047519

**Published:** 2026-01-30

**Authors:** Wenjie Zhang, Anqi Wei, Guanghua Xie

**Affiliations:** a Department of Gastrointestinal Surgery, Heze Municipal Hospital, Heze, Shandong Province, China; b Modern Educational Technology Center, Heze Medical College, No. 5025 Taian Road, Heze, Shandong Province, China; c Department of Hepatobiliary Surgery, Yanbian University Hospital, Yanji, Jilin Province, China.

**Keywords:** clinical risk prediction model, nomogram, post-endoscopic retrograde cholangiopancreatography cholangitis, risk factors

## Abstract

Research on risk factors and predictive models for post-endoscopic retrograde cholangiopancreatography (post-ERCP) cholangitis remains limited. This study aimed to identify key risk factors for post-ERCP cholangitis and develop a clinical risk prediction model to enhance risk assessment accuracy and provide a reliable basis for clinical decision-making. The study cohort was randomly divided into a training set and an validation set at a ratio of 7:3. Within the training set, independent risk factors for post-ERCP cholangitis were screened through univariate analysis, LASSO regression, and subsequent multivariate logistic regression. A predictive nomogram was then constructed based on the identified risk factors. The performance of this nomogram was assessed in both the training and validation sets by receiver operating characteristic curves, calibration curves, the area under the curve, and the Hosmer–Lemeshow (H–L) test. Furthermore, its potential clinical utility was evaluated using decision curve analysis and clinical impact curve. A predictive nomogram was developed incorporating the following independent risk factors: history of diabetes mellitus (OR 3.698, 95% CI [1.734–7.962], *P* <.001), previous ERCP (OR 2.451, 95% CI [1.079–5.484], *P *= .03), malignant biliary obstruction (OR 2.750, 95% CI [1.185–6.375], *P* = .018), high biliary obstruction (OR 3.343, 95% CI [1.394–7.987], *P* = .006), and albumin <35g/L (OR 5.499, 95% CI 2.493–12.496], *P* <.001). The prediction models based on these factors achieved an area under the curve of 0.903 (95%CI:0.85–0.947) in the training set and 0.884 (95% CI:0.803–0.966) in the validation set. The calibration curve shows strong alignment between model predictions and actual outcomes. The *P*-values from the H–L test were .845 for the training set and .121 for the validation set, indicating no significant deviation between predicted and actual values. decision curve analysis demonstrated that the model offered a significant net clinical benefit across a broad range of risk thresholds. Clinical impact curve showed that at specific thresholds, the model’s identification of high-risk patients was highly consistency with actual outcomes, confirming its clinical utility. The nomogram for predicting post-ERCP cholangitis, based on identified risk factors, demonstrated excellent predictive performance and clinical utility. Therefore, it can assist clinicians in early identification of high-risk patients and in developing personalized intervention strategies.

## 1. Introduction

Endoscopic retrograde cholangiopancreatography (ERCP), first reported by McCune^[1]^ in 1968, has become a key technique for diagnosing and treating biliary and pancreatic diseases. Initially used for diagnosis,^[2]^ ERCP has since been expanded to treat benign biliary and pancreatic lesions, tumors, and postoperative complications, among other complex conditions. Compared with traditional surgical procedures, ERCP offers advantages such as reduced trauma, increased safety, and faster postoperative recovery. However, ERCP is an invasive procedure that is technically challenging and complex, potentially leading to complications such as postoperative pancreatitis, cholangitis, cholecystitis, bleeding, and perforation.^[3]^ Post-ERCP cholangitis is one of the most common and severe complications. The clinical manifestations include fever, jaundice, and abdominal pain. In severe cases, it can trigger sepsis, infectious shock, and systemic inflammatory response syndrome, potentially leading to death with a mortality rate exceeding 10%.^[3,4]^ Therefore, predicting the risk of post-ERCP cholangitis and identifying associated risk factors are crucial.

Several studies have identified risk factors for post-ERCP cholangitis. Yilmaz et al reported that malignant biliary obstruction (MBO), high body mass index (BMI), and prolonged surgery increase the risk.^[5]^ Chen Min et al found that age >60 years, previous ERCP history, and hepatic hilar obstruction are independent risk factors, while bile stone extraction offers a protective effect.^[6]^ Tierny et al associated MBO, multiple stents, and hypoproteinemia with a higher likelihood of postoperative cholangitis, suggesting that regular replacement of biliary stents may reduce long-term cholangitis.^[7]^ However, most studies have focused on a single disease or a narrow range of variables. A comprehensive risk assessment and prediction model for post-ERCP cholangitis is still lacking, along with effective tools to assist clinicians in making accurate risk assessments and clinical decisions. Therefore, this study aimed to identify the key risk factors of post-ERCP cholangitis and develop a clinical risk prediction model that improves risk assessment accuracy, providing clinicians with a reliable decision-making tool.

## 2. Methods

### 2.1. Study population

This study was approved by the Ethics Committee of Yanbian University Hospital (Approval No. 2024392). We retrospectively analyzed the clinical data of patients who underwent ERCP at the Department of Hepatobiliary Surgery, Yanbian University Hospital, between June 2020 and June 2024. The inclusion criteria were as follows: age ≥18 years, patients who met the indications for ERCP and underwent the procedure, successful completion of the ERCP procedure, availability of complete pre-procedural, procedural, and post-procedural clinical data for analysis. The exclusion criteria were as follows: suspected or confirmed cholangitis before ERCP, existing pancreatitis before ERCP, other concurrent serious infections, surgical failure or conversion to open surgery, history of other surgeries within 1 month before ERCP, and incomplete clinical data. This study followed the STROCSS guidelines^[8]^ and was approved by the institutional review board and conducted in accordance with the ethical guidelines of the World Medical Association Declaration of HELSINKI.

### 2.2. Diagnostic criteria and relevant definitions

(1) According to the“ERCP-related adverse events, European Society of Gastrointestinal Endoscopy guideline”^[3]^ and the“Tokyo Guidelines 2018,”^[9]^ the following criteria defined post-ERCP cholangitis: absence of fever before ERCP, with postoperative fever (>38.0°C) lasting over 24 hours or abnormal leukocyte counts (>10.0 × 10^9^/L or <4.0 × 10^9^/L); worsening jaundice or epigastric pain, with elevated cholestasis-related biochemical markers; exclusion of other systemic infections.
(2) Difficulty in intubation was defined as an intubation time exceeding 10 minutes or failure to successfully access the bile duct after more than 5 attempts with a guidewire.
(3) MBO is a condition caused by malignant tumors and is characterized by biliary strictures and impaired bile drainage.^[10]^ In contrast, benign biliary obstruction involves nonmalignant lesions (e.g., stones and inflammatory strictures) that obstruct the biliary system, leading to symptoms such as jaundice and abdominal pain.
(4) Obstruction sites were classified as high or low. High bile duct obstruction occurs above the confluence of the cystic and common bile ducts, while low bile duct obstruction occurs below this junction.

### 2.3. Endoscopic procedures for ERCP

During ERCP, oral dacronin hydrochloride colloidal syrup was administered prior to the procedure to achieve pharyngeal surface anesthesia, thereby reducing patient discomfort as well as salivary and respiratory secretions, followed by intravenous sedation with propofol and dexmedetomidine. The oxygen saturation, blood pressure, respiratory rate, and heart rate were monitored continuously. With the patient in the left-prone position, the duodenoscope was inserted orally and advanced through the esophagus and stomach into the descending duodenum to locate the duodenal papilla. A guide wire was inserted into the bile duct using an incision knife. If unsuccessful after multiple attempts or prolonged time, pre-incision of the duodenal papillary muscle or the double-guidewire technique may be considered. Depending on the situation, cholangiography, endoscopic papillary sphincterotomy, endoscopic papillary balloon dilatation, endoscopic retrograde biliary stent drainage, endoscopic nasobiliary drainage, or radiofrequency ablation may be performed during the procedure.

### 2.4. Data collection

To ensure data quality, all clinical data were independently reviewed and verified by 2 researchers. A trial administrator was then responsible for data consolidation and quality control. Patients were screened based on inclusion and exclusion criteria. All clinical data were carefully recorded. General information and medical history: age, sex, BMI, smoking history, alcohol use, diabetes mellitus, history of hypertension, hepatitis, cirrhosis, gastrointestinal surgeries (e.g., cholecystectomy, choledochotomy, appendectomy), and previous ERCP; preoperative examinations and laboratory tests: white blood cell count (WBC), hemoglobin (Hb), platelet count, total bilirubin (T-BIL), direct bilirubin (D-BIL), alanine aminotransferase, aspartate aminotransferase, alkaline phosphatase (ALP), γ-glutamyltransferase, total protein (TP), albumin (ALB), bile duct sizes, endoscopic sphincterotomy (EST), type of implanted stent and site of obstruction. Additional factors: gallstones, cause of biliary obstruction, presence of periampullary diverticulum, total procedure time, difficult cannulation, and number of stents implanted. Bile duct sizes were assessed by 2 clinicians using magnetic resonance cholangiopancreatography or biliary thin-layer computed tomography, and the results were reviewed and confirmed by a third physician.

### 2.5. Sample size consideration

Based on the published literature, particularly the guideline “ERCP-related adverse events: European Society of Gastrointestinal Endoscopy Guideline” (Endoscopy),^[3]^ which reports that the incidence of post-ERCP cholangitis is approximately 3%, we estimated the required sample size for developing our predictive model. The model incorporated 5 independent predictor variables. In accordance with established sample size calculation methods for prediction model development, the minimum required sample size was estimated to be 377. Our final cohort meets this requirement.

### 2.6. Statistical analyses

Statistical analysis was performed using R software (version 4.3.3; R Foundation for Statistical Computing, Vienna, Austria). Normally distributed variables were presented as mean ± standard deviation (x ± s) and compared using the *t*-test. Non-normally distributed variables were expressed as median (interquartile range) and compared using the Mann–Whitney *U*-test. Categorical variables were shown as numbers and percentages, with comparisons made using Pearson chi-square test or Fisher exact test. Patients were randomly categorized into training and validation sets in a 7:3 ratio. Variables in the training set were analyzed using univariate analysis with a significance threshold of *P* <.05. Significant variables were further screened using LASSO regression, and those selected based on lambda.min were analyzed using multivariate logistic regression (*P*<.05). A clinical risk prediction model was developed based on the multivariate logistic regression results. A nomogram was constructed to visualize the contribution of each variable to the risk prediction. The model performance and calibration were assessed by generating receiver operating characteristic and calibration curves for both the training and validation sets, with the area under the curve calculated. The model’s fit was further evaluated using the Hosmer–Lemeshow (H–L) goodness-of-fit test. Finally, decision curve analysis (DCA) and clinical impact curves (CIC) were used to comprehensively evaluate the clinical utility of the model.

## 3. Results

### 3.1. Baseline data

**Figure 1.**
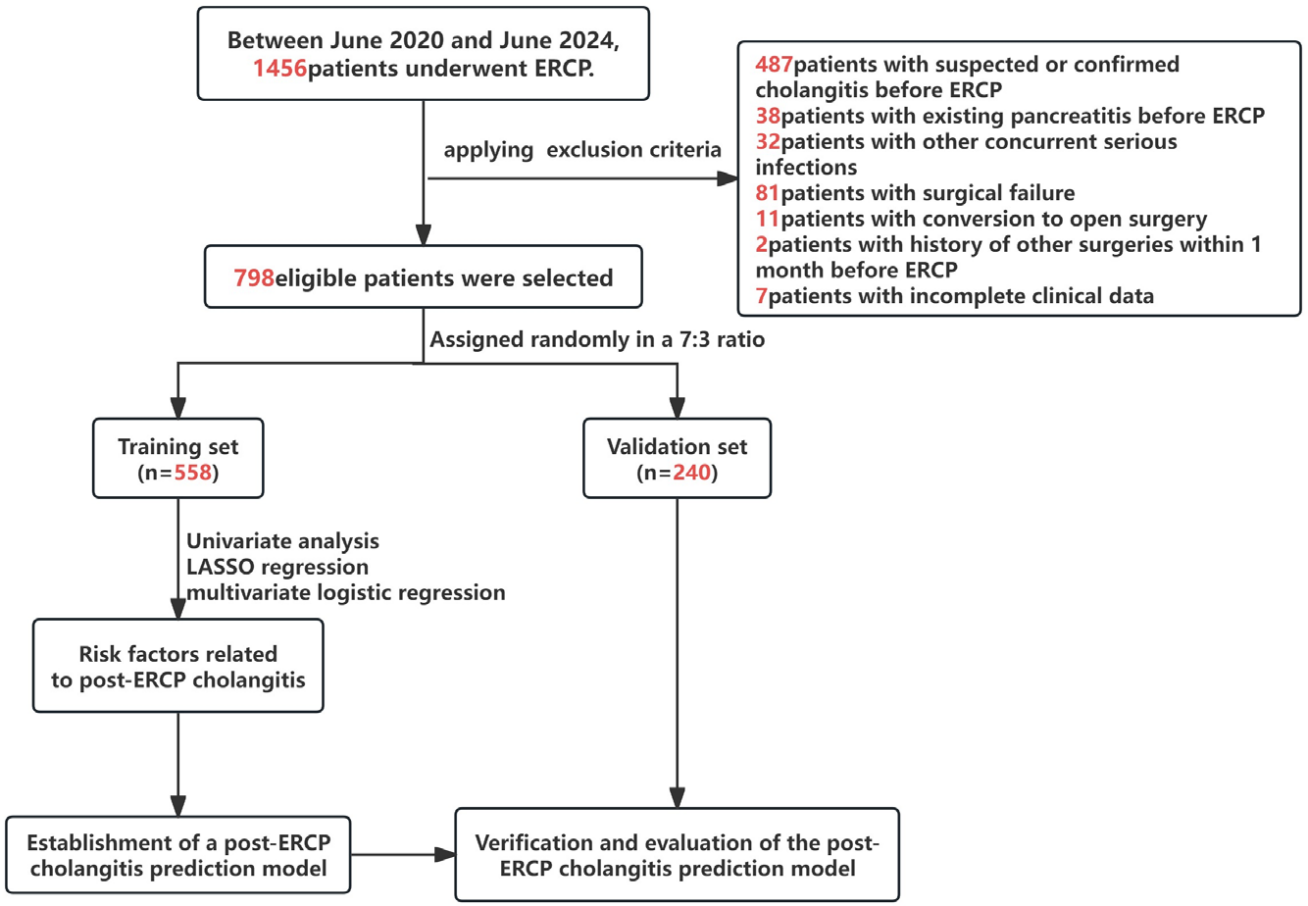
Flowchart of the study.

Between June 2020 and June 2024, 1456 patients underwent ERCP at the Department of Hepatobiliary Surgery, Yanbian University Hospital. After applying the inclusion and exclusion criteria, 798 eligible patients were selected, of whom 67 (8.3%) developed postoperative cholangitis. The patients were randomly assigned to a training set (558 patients, 48 of whom developed cholangitis) and a validation set (240 patients, 19 of whom developed cholangitis) in a 7:3 ratio. The screening process is detailed in the flowchart (Fig. 1). No statistically significant differences were found in the characteristics of the training and validation sets (*P* >.05, Table 1), indicating good comparability between the 2 groups.

**Table 1:** The baseline characteristics of the training and validation sets.

Characteristics	Training set (n = 558)	Validation set (n = 240)	χ^2^/Z	*P*
Age (years, M [IQR])	70.0 (60.0;77.0)	71.0 (59.8;76.0)	0.234	.815
Sex (n [%])				
Male	295 (52.9%)	127 (52.9%)	<0.001	1
Female	263 (47.1%)	113 (47.1%)		
BMI (kg/m^2^, M [IQR])	23.2 (20.9; 25.5)	23.5 (21.4; 25.6)	1.296	.195
Smoking history (n (%])				
No	394 (70.6%)	177 (73.8%)	0.666	.414
Yes	164 (29.4%)	63 (26.2%)		
Alcohol use (n [%])				
No	439 (78.7%)	176 (73.3%)	2.414	.12
Yes	119 (21.3%)	64 (26.7%)		
Diabetes mellitus (n [%])				
No	444 (79.6%)	197 (82.1%)	0.521	.47
Yes	114 (20.4%)	43 (17.9%)		
History of hypertension (n [%])				
No	342 (61.3%)	146 (60.8%)	0.002	.966
Yes	216 (38.7%)	94 (39.2%)		
History of hepatitis (n [%])				
No	517 (92.7%)	222 (92.5%)	<0.001	1
Yes	41 (7.35%)	18 (7.50%)		
History of cirrhosis (n [%])				
No	554 (99.3%)	237 (98.8%)	0.107	.744
Yes	4 (0.72%)	3 (1.25%)		
History of gastrointestinal surgery (n [%])				
No	347 (62.2%)	158 (65.8%)	0.81	.368
Yes	211 (37.8%)	82 (34.2%)		
Previous ERCP (n [%])				
No	465 (83.3%)	195 (81.2%)	0.374	.541
Yes	93 (16.7%)	45 (18.8%)		
Gallstones (n [%])				
No	266 (47.7%)	99 (41.2%)	2.535	.111
Yes	292 (52.3%)	141 (58.8%)		
Cause of biliary obstruction (n [%])				
BBO	447 (80.1%)	185 (77.1%)	0.757	.384
MBO	111 (19.9%)	55 (22.9%)		
Bile duct sizes (mm, M [IQR])	10.1 (7.70; 13.0)	9.95 (7.70;13.2)	0.056	.956
Site of obstruction (n [%])				
Low bile duct obstruction	495 (88.7%)	204 (85.0%)	1.798	.18
High bile duct obstruction	63 (11.3%)	36 (15.0%)		
WBC (×109/L, M [IQR])	6.47 (5.17; 7.99)	6.39 (5.12; 7.88)	0.459	.646
Hb (g/L, M [IQR])	128 (118; 139)	128 (115; 140)	0.471	.638
Platelet [×109/L, M (IQR) ]	208 (170; 261)	221 (177; 274)	1.677	.094
T-BIL (µmol/L, M [IQR])	31.6 (14.7; 81.7)	30.2 (15.0; 82.0)	0.24	.81
D-BIL (µmol/L, M [IQR])	19.5 (6.70;62.8)	19.9 (6.52; 66.8)	0.123	.902
AST (U/L, M [IQR])	58.5 (23.0;130)	46.5 (22.0; 124)	0.698	.485
ALT (U/L, M (IQR])	78.0 (29.0; 162)	61.0 (23.8; 155)	1.021	.307
ALP (U/L, M [IQR])	182 (100; 300)	180 (102; 308)	0.275	.783
GGT (U/L, M [IQR])	284 (93.2; 587)	298 (90.5; 637)	0.999	.318
ALB (n [%])				
≥ 35 g/L	448 (80.3%)	194 (80.8%)	0.007	.935
<35 g/L	110 (19.7%)	46 (19.2%)		
TP (n [%])				
≥60 g/L	391 (70.1%)	171 (71.2%)	0.062	.803
<60 g/L	167 (29.9%)	69 (28.7%)		
Total procedure time (min, M [IQR])	45.0 (35.0; 65.0)	50.0 (35.0; 60.2)	0.162	.871
Periampullary diverticulum (n [%])				
No	453 (81.2%)	199 (82.9%)	0.231	.63
Yes	105 (18.8%)	41 (17.1%)		
Difficult cannulation (n [%])				
No	447 (80.1%)	191 (79.6%)	0.005	.942
Yes	111 (19.9%)	49 (20.4%)		
Number of stents implanted (n [%])				
0	424 (76.0%)	178 (74.2%)	2.478	.29
1	83 (14.9%)	45 (18.8%)		
2	51 (9.14%)	17 (7.08%)		

ALB = albumin, ALP = alkaline phosphatase, ALT = alanine aminotransferase, AST = aspartate aminotransferase, BBO = benign biliary obstruction, BMI = body mass index, D-BIL = direct bilirubin, GGT = γ-glutamyltransferase, Hb = hemoglobin, MBO = Malignant biliary obstruction, T-BIL = total bilirubin, TP = total protein, WBC = white blood cell count.

### 3.2. Risk factors related to post-ERCP cholangitis in the training set

**Figure 2.**
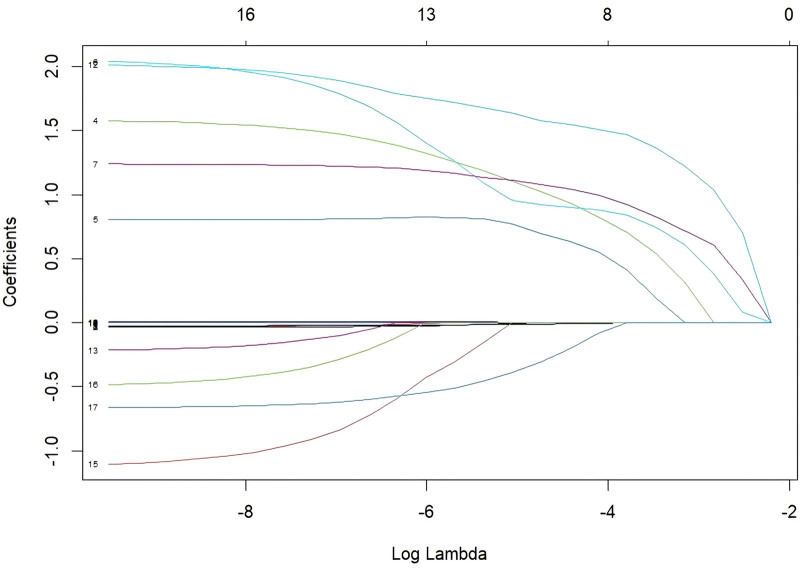
LASSO path plot. The trajectory of the regression coefficients is shown as the regularization parameter λ changes. As λ increases, some coefficients shrink to zero, facilitating variable selection.

**Figure 3.**
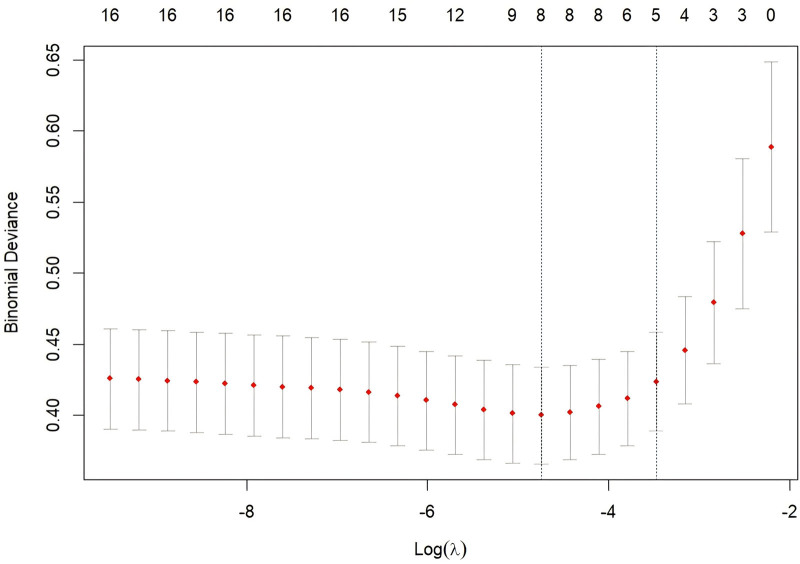
Cross-validation curve. Using 10-fold cross-validation, Lambda.min = 0.00871062 (left), representing the lowest cross-validation error, provides the best prediction performance and retains the most key variables. In contrast, Lambda.1se = 0.03102939 (right) is 1 standard error from the optimal point, discarding more features and further reducing the risk of overfitting.

**Figure 4.**
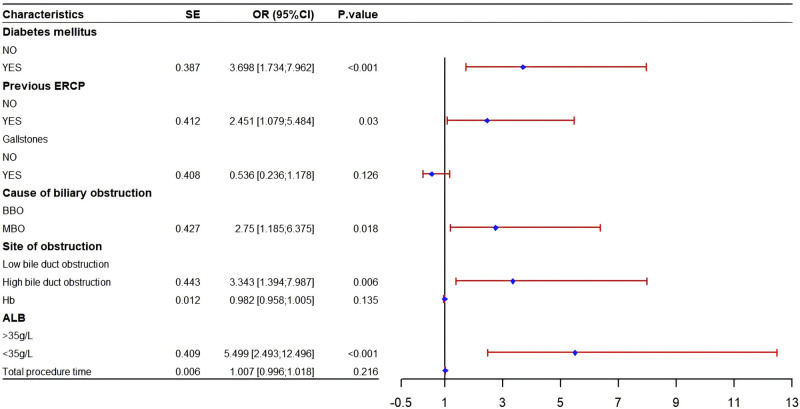
Forest plot of multivariable analysis. BBO, MBO. Diabetes mellitus (OR 3.698, 95% CI [1.734–7.962], *P* <.001), previous ERCP (OR 2.451, 95% CI [1.079–5.484], *P* = .03), malignant biliary obstruction (OR 2.75, 95% CI [1.185–6.375], *P* = .018), high bile duct obstruction (OR 3.343, 95% CI [1.394–7.987], *P* = .006), and ALB <35 g/L (OR 5.499, 95% CI [2.493–12.496], *P* <.001). ALB = albumin, BBO = benign biliary obstruction, ERCP = endoscopic retrograde cholangiopancreatography, MBO = malignant biliary obstruction, OR =odds ratio.

Univariate analysis identified 15 variables significantly associated with post-ERCP cholangitis (*P* <.05), including age, BMI, diabetes mellitus, previous ERCP, gallstones, MBO, high bile duct obstruction, Hb, T-BIL, D-BIL, ALP, ALB <35 g/L, TP <60 g/L, total procedure time, and number of stents implanted (Table 2). LASSO regression was applied to these significant variables for dimensionality reduction. Cross-validation determined the optimal model parameters, with lambda.min (λ = 0.00871062) chosen. This approach minimized covariance and avoided overfitting while preserving statistically significant variables. LASSO regression identified 8 key variables: diabetes mellitus, previous ERCP, gallstones, MBO, high bile duct obstruction, Hb, ALB <35 g/L, and total procedure time (Figs. 2 and 3). Multifactorial logistic regression identified significant associations for diabetes mellitus (OR 3.698, 95% CI [1.734–7.962], *P* <.001), previous ERCP (OR 2.451, 95% CI [1.079–5.484], *P* = .03), MBO (OR 2.75, 95% CI [1.185–6.375], *P* = .018), high bile duct obstruction (OR 3.343, 95% CI [1.394–7.987], *P* = .006), and ALB <35 g/L (OR 5.499, 95% CI [2.493–12.496], *P* <.001) (Fig. 4).

**Table 2:** Univariate analysis for predictors of post-ERCP cholangitis.

Characteristics	Non-cholangitis (n = 510)	Cholangitis (n = 48)	OR (95% CI)	Z	*P*
Age (year, M [IQR])	69.5 (59.0; 77.0)	73.0 (66.0; 79.5)	1.030 (1.006; 1.056)	2.342	.019
Sex (n [%])					
Male	271 (53.1%)	24 (50.0%)			
Female	239 (46.9%)	24 (50.0%)	1.134 (0.625; 2.056)	0.416	.677
BMI [kg/m2, M (IQR) ]	23.3 (21.1; 25.7)	21.5 (20.0; 24.7)	0.898 (0.820; 0.978)	−2.365	.018
Smoking history (n [%])					
No	359 (70.4%)	35 (72.9%)			
Yes	151 (29.6%)	13 (27.1%)	0.883 (0.439; 1.677)	−0.367	.714
Alcohol use (n [%])					
No	397 (77.8%)	42 (87.5%)			
Yes	113 (22.2%)	6 (12.5%)	0.502 (0.188; 1.126)	−1.534	.125
Diabetes mellitus (n [%])					
No	422 (82.7%)	22 (45.8%)			
Yes	88 (17.3%)	26 (54.2%)	5.667 (3.077; 10.538)	5.551	<.001
History of hypertension (n [%])					
No	311 (61.0%)	31 (64.6%)			
Yes	199 (39.0%)	17 (35.4%)	0.857 (0.453; 1.571)	−0.49	.624
History of hepatitis (n [%])					
No	474 (92.9%)	43 (89.6%)			
Yes	36 (7.06%)	5 (10.4%)	1.531 (0.506;3.788)	0.847	.397
History of cirrhosis (n [%])					
No	507 (99.4%)	47 (97.9%)			
Yes	3 (0.59%)	1 (2.08%)	3.596 (0.176; 28.713)	1.099	.272
History of gastrointestinal surgery (n [%])					
No	315 (61.8%)	32 (66.7%)			
Yes	195 (38.2%)	16 (33.3%)	0.808 (0.422; 1.489)	−0.669	.504
Previous ERCP (n [%])					
No	435 (85.3%)	30 (62.5%)			
Yes	75 (14.7%)	18 (37.5%)	3.48 (1.820; 6.508]	3.857	<.001
Gallstones (n [%])					
No	233 (45.7%)	33 (68.8%)			
Yes	277 (54.3%)	15 (31.2%)	0.382 (0.197;0.709)	−2.969	.003
Cause of biliary obstruction (n [%])					
BBO	430 (84.3%)	17 (35.4%)			
MBO	80 (15.7%)	31 (64.6%)	9.801 (5.242;18.910)	7.014	<.001
Bile duct sizes (mm, M [IQR])	9.90 (7.70;13.0)	11.2 (8.43;14.1)	1.054 (0.986;1.124)	1.588	.112
Site of obstruction (n [%])					
Low bile duct obstruction	470 (92.2%)	25 (52.1%)			
High bile duct obstruction	40 (7.84%)	23 (47.9%)	10.810 (5.628; 20.841)	7.158	<.001
WBC (×10^9^/L, M [IQR])	6.41 (5.13; 7.93)	7.00 (5.65; 8.00)	1.100 (0.923; 1.310)	1.077	.282
Hb (g/L, M [IQR])	129 (119;139)	116 (103; 125)	0.951 (0.933; 0.969)	−5.334	<.001
Platelet (×10^9^/L, M [IQR])	208 (170; 259)	228 (158; 300)	1.003 (0.999; 1.007)	1.524	.128
T-BIL (µmol/L, M [IQR])	30.4 (14.2; 76.8)	61.8 (21.1; 202)	1.005 (1.002; 1.007)	3.897	<.001
D-BIL (µmol/L, M [IQR])	18.9 (6.40; 59.5)	49.7 (14.8; 160)	1.006 (1.003; 1.009)	4.204	<.001
AST (U/L, M [IQR])	56.0 (23.0; 128)	79.5 (32.0; 147)	1.001 (0.997; 1.002)	0.051	.959
ALT (U/L, M [IQR])	78.0 (27.2; 164)	88.5 (35.8; 148)	0.999 (0.996; 1.001)	−0.613	.54
ALP (U/L, M [IQR])	173 (99.0; 282)	322 (168; 464)	1.001 (1.001; 1.002)	2.934	.003
GGT (U/L, M [IQR])	280 (79.2; 581)	392 (220; 643)	1.001 (0.999; 1.002)	1.872	.061
ALB [n (%) ]					
≥ 35 g/L	434 (85.1%)	14 (29.2%)	–	–	–
<35 g/L	76 (14.9%)	34 (70.8%)	13.868 (7.252; 27.856)	7.711	<.001
TP (n [%])					
≥60 g/L	370 (72.5%)	21 (43.8%)	–	–	–
<60 g/L	140 (27.5%)	27 (56.2%)	3.398 (1.866; 6.268]	3.979	<.001
Total procedure time (min, M [IQR])	45.0 (35.0; 65.0)	55.0 (40.0; 82.5)	1.010 (1.002; 1.018)	2.417	.016
Periampullary diverticulum (n [%])					
No	417 (81.8%)	36 (75.0%)			
Yes	93 (18.2%)	12 (25.0%)	1.495 (0.721; 2.906)	1.14	.263
Difficult cannulation (n [%])					
No	410 (80.4%)	37 (77.1%)			
Yes	100 (19.6%)	11 (22.9%)	1.219 (0.575; 2.399)	0.548	.584
Number of stents implanted [n (%) ]					
0	405 (79.4%)	19 (39.6%)	–	–	–
1	64 (12.5%)	19 (39.6%)	6.328 (3.169; 12.666)	5.253	<.001
2	41 (8.04%)	10 (20.8%)	5.199 (2.193; 11.738)	3.891	<.001

ALB = albumin, ALP = alkaline phosphatase, ALT = alanine aminotransferase, AST = aspartate aminotransferase, BBO = benign biliary obstruction, BMI = body mass index, D-BIL = direct bilirubin, GGT = γ-glutamyltransferase, Hb = hemoglobin, MBO = Malignant biliary obstruction, OR = odds ratio, T-BIL = total bilirubin, TP = total protein, WBC = white blood cell count.

### 3.3. Establishment and verification of the nomogram prediction model

**Figure 5.**
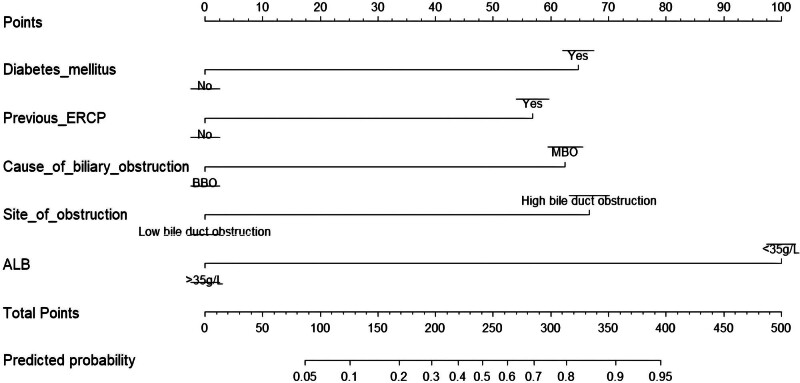
Nomogram for prediction of post-ERCP cholangitis. Each factor on the nomogram corresponds to a specific score based on the regression coefficients obtained from the analysis. The overall score for each patient is calculated by summing the individual scores of all independent risk factors. Clinicians can accurately determine the predicted probability of postoperative cholangitis by referencing the overall score on the nomogram. ERCP = endoscopic retrograde cholangiopancreatography.

A binary logistic regression model predicting post-ERCP cholangitis was constructed based on 5 independent risk factors: diabetes mellitus, previous ERCP, MBO, high bile duct obstruction, and ALB level <35 g/L. This model was visualized using a nomogram (Fig. 5).

**Figure 6.**
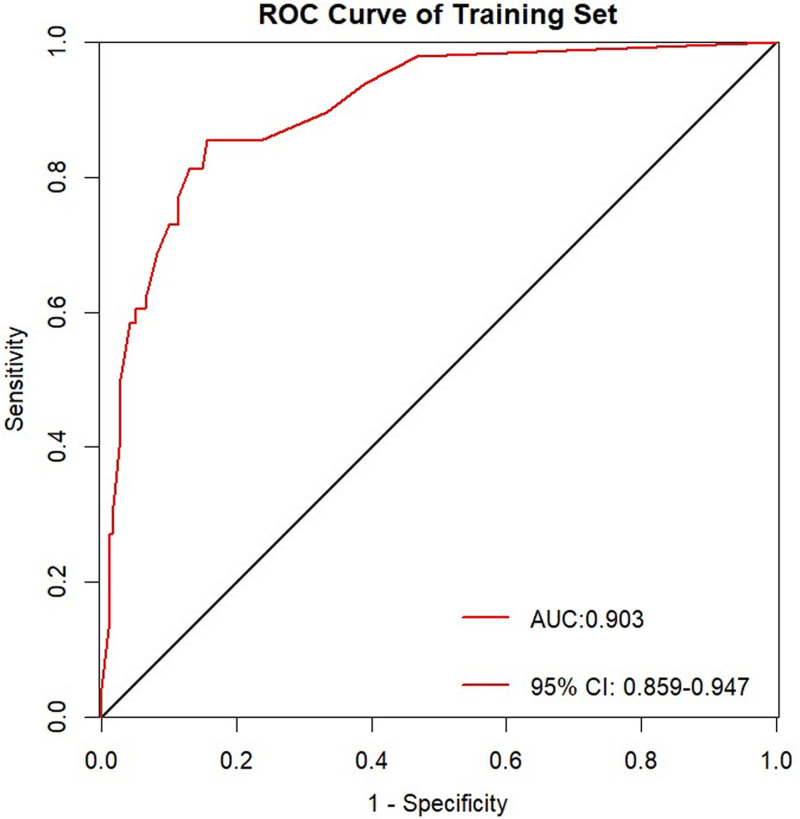
ROC curve of the training set. ROC = receiver operating characteristic.

**Figure 7.**
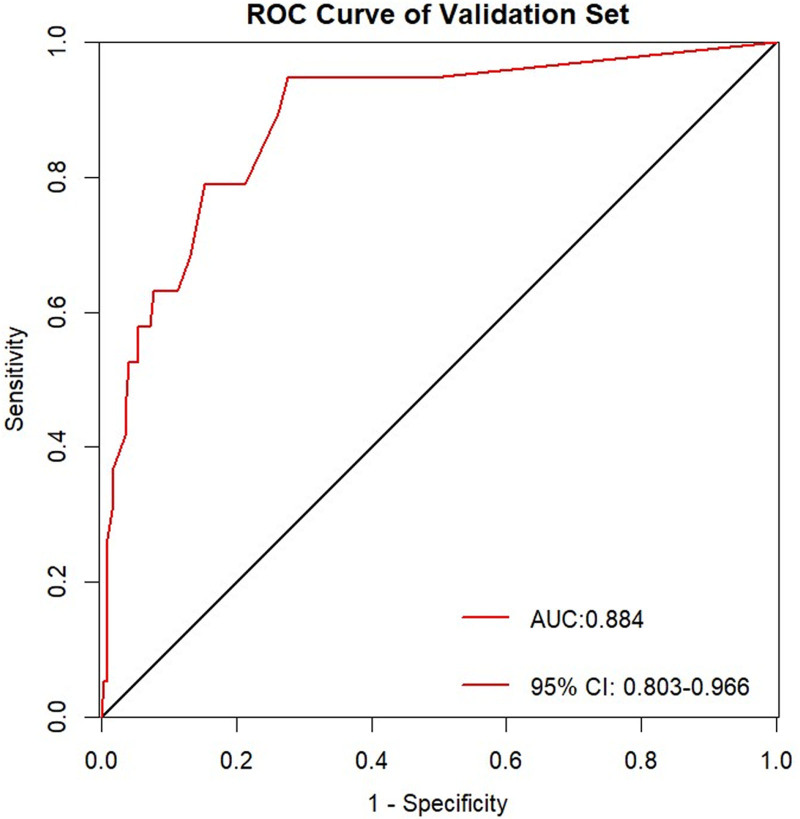
ROC curve of the validation set. ROC = receiver operating characteristic.

**Figure 8.**
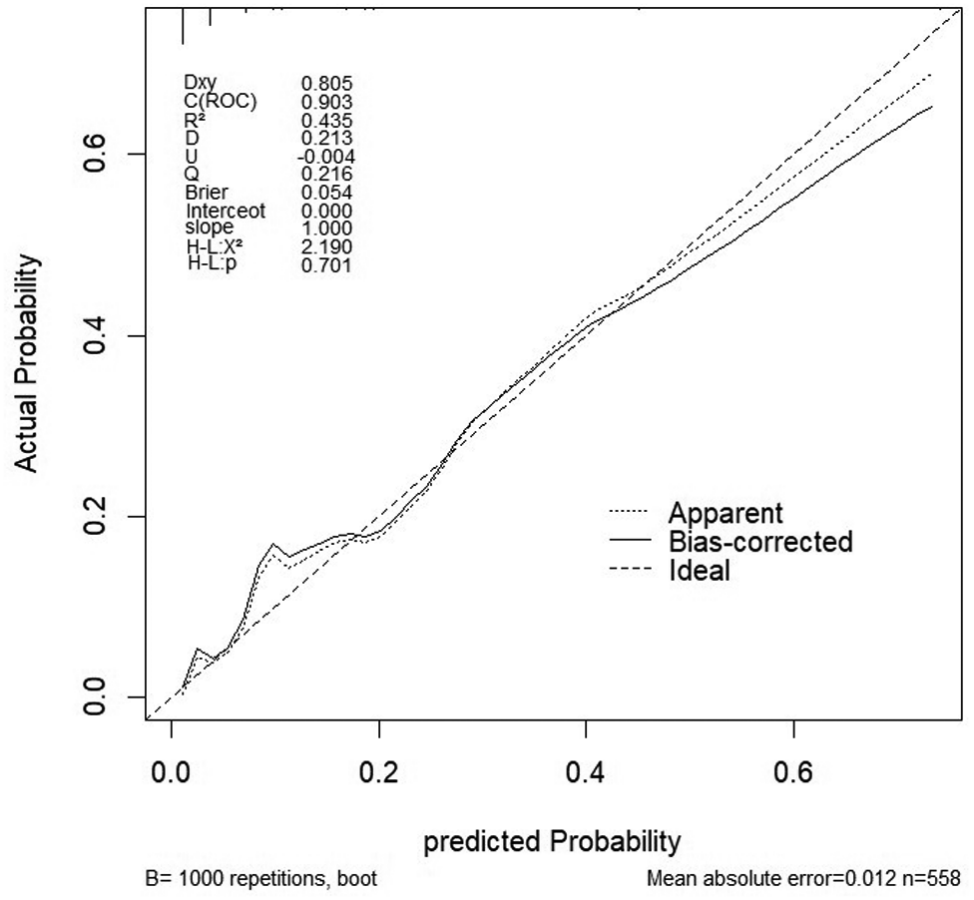
Calibration curve of training set. Apparent indicates the prediction curve without calibration; Bias-corrected indicates the calibrated prediction curve; Ideal represents the standard curve.

**Figure 9.**
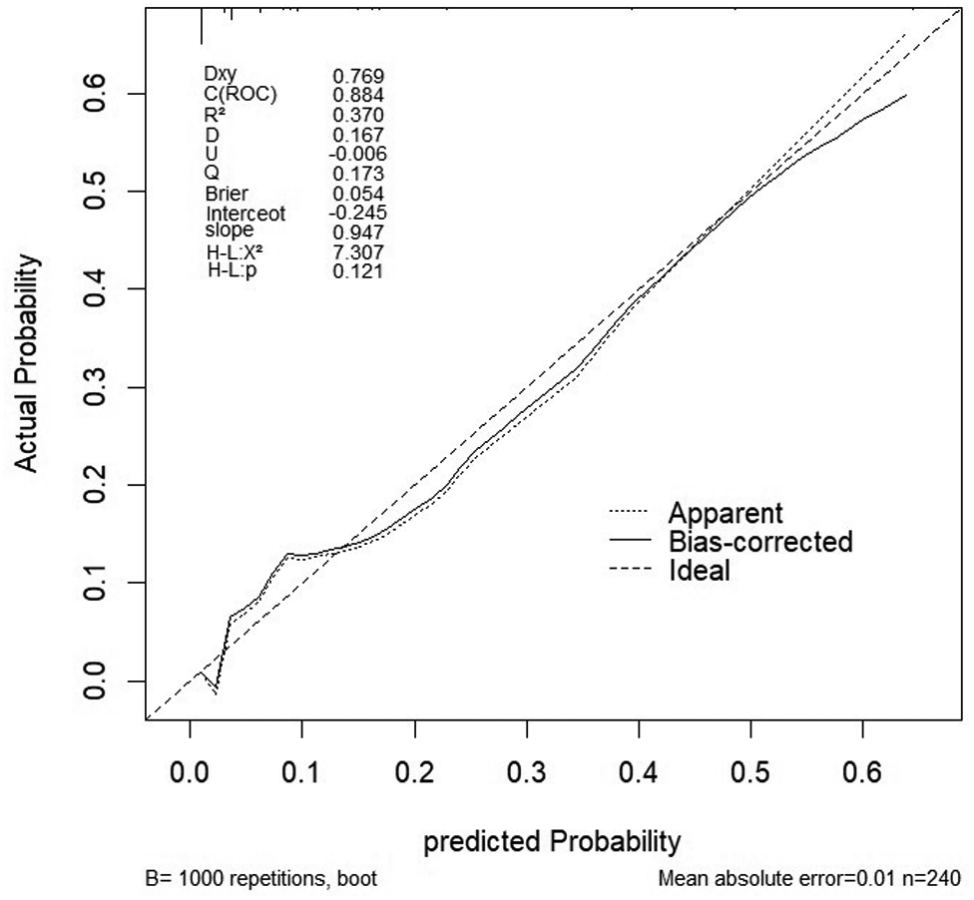
Calibration curve of validation set. Apparent indicates the prediction curve without calibration; bias-corrected indicates the calibrated prediction curve; ideal represents the standard curve.

The performance of the model was evaluated using receiver operating characteristic curves, with an area under the curve of 0.903 (95% CI:0.859–0.947) for the training set and 0.884 (95% CI:0.803–0.966) for the validation set, indicating excellent discriminative ability in both sets (Figs. 6 and 7). Using 1000 bootstrap resamples, the calibration curves demonstrated strong agreement between the model predictions and actual outcomes (Figs. 8 and 9). In the training set, the average absolute error was 0.012, the mean square error was 0.00045, the Dxy value was 0.805, the R^2^ value was 0.435, and the brier score was 0.054, indicating strong predictive accuracy and consistency. In the validation set, the average absolute error was 0.009, the mean square error was 0.00023, the Dxy value was 0.769, the R^2^ value was 0.370, and the brier score was 0.054, suggesting a similar predictive performance for the new data. Additionally, the H-L goodness-of-fit test yielded X^2^ = 2.189 (*P* = .845) for the training set and X^2^ = 7.3066 (*P* = .121) for the validation set. With *P*-values >.05, the model fitted well in both sets and showed no significant deviation from the actual observations.

**Figure 10.**
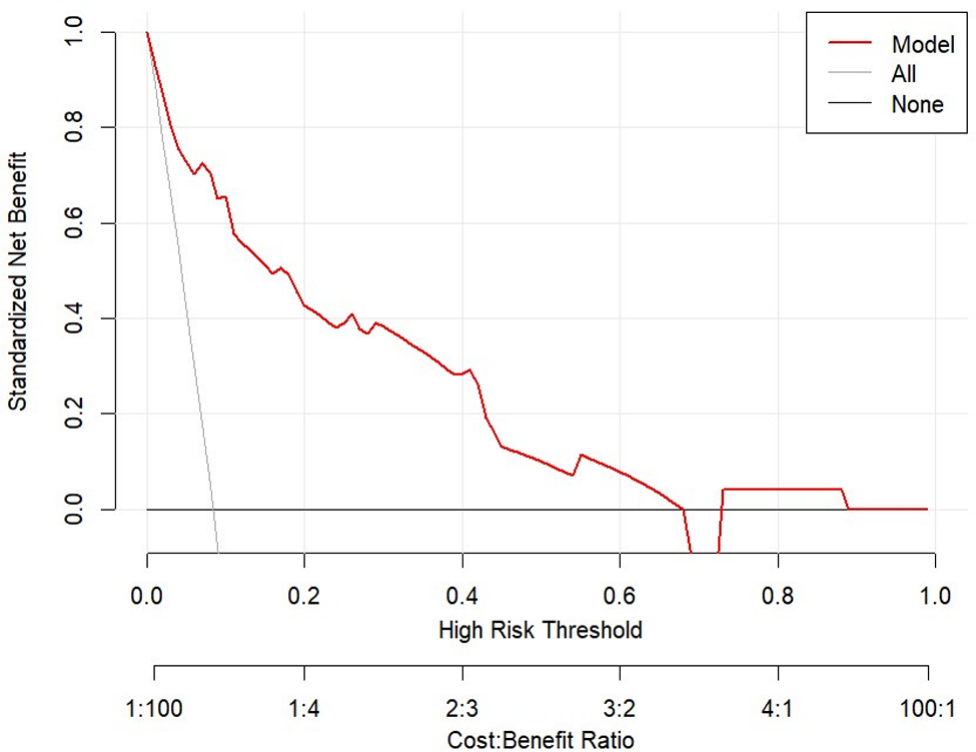
DCA curve for the training set. (none no intervention, all full intervention). In the training set, the model’s net benefit was significantly higher than “no intervention” and “all intervention” strategies at thresholds between 1 % and 68% and 73% to 88%. DCA = decision curve analysis.

**Figure 11.**
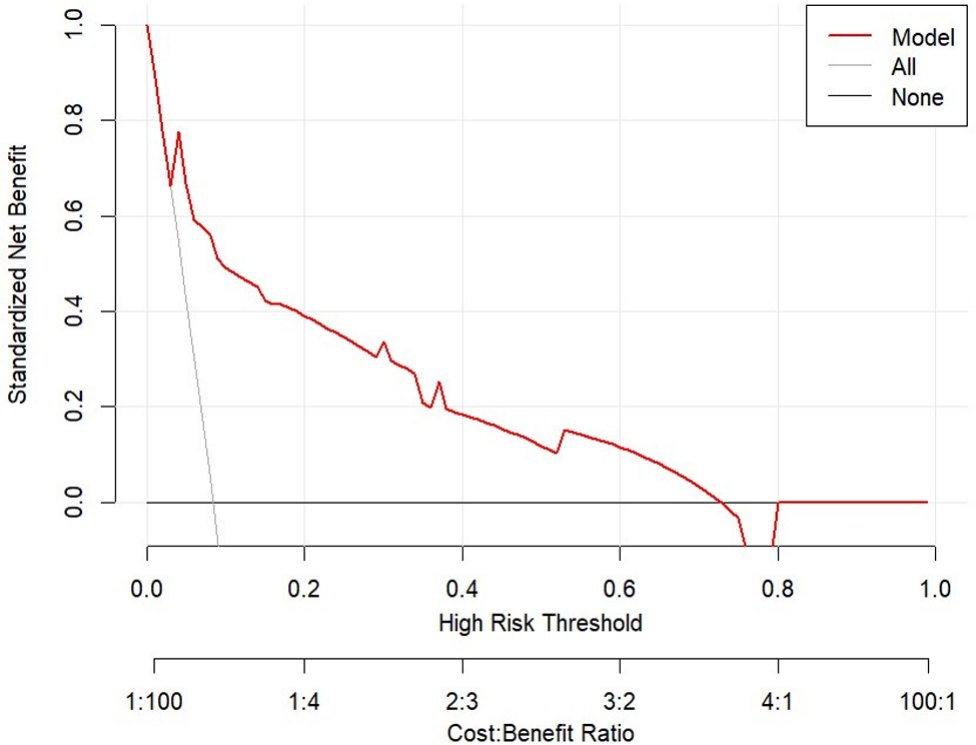
DCA curve for the validation set. (none no intervention, all full intervention). In the validation set, the model’s net benefit was significantly higher than “no intervention” and “all intervention” strategies at thresholds of 4% to 72%. DCA = decision curve analysis.

**Figure 12.**
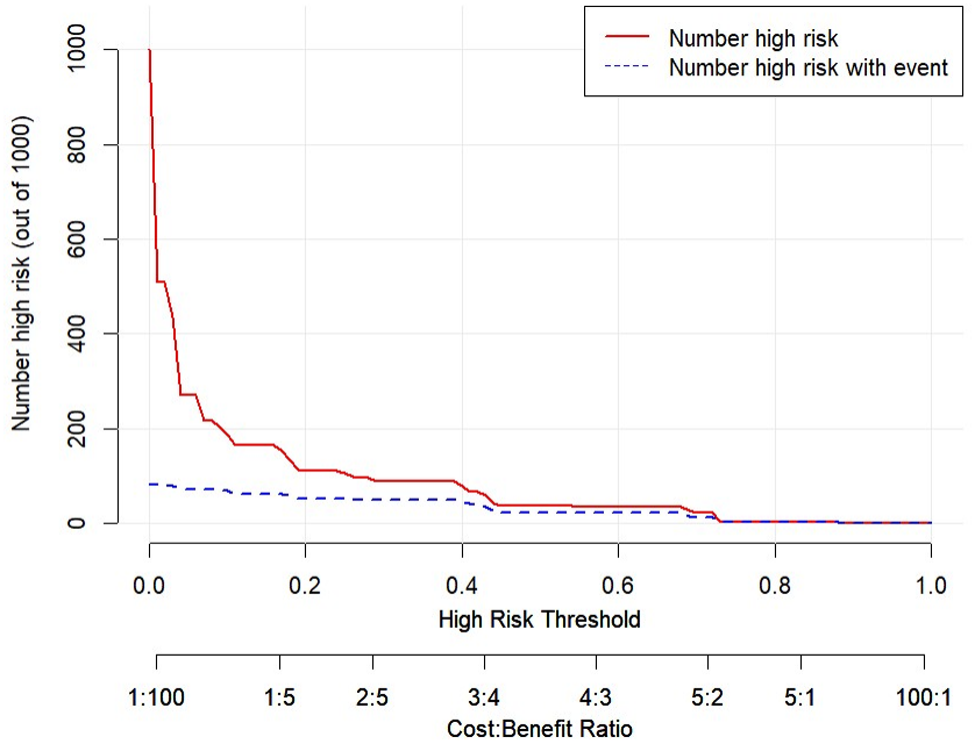
CIC curve for the training set. In the training set, when the threshold probability exceeded 42%, the high-risk predictions of the model closely matched the actual cases. CIC = clinical impact curve.

**Figure 13.**
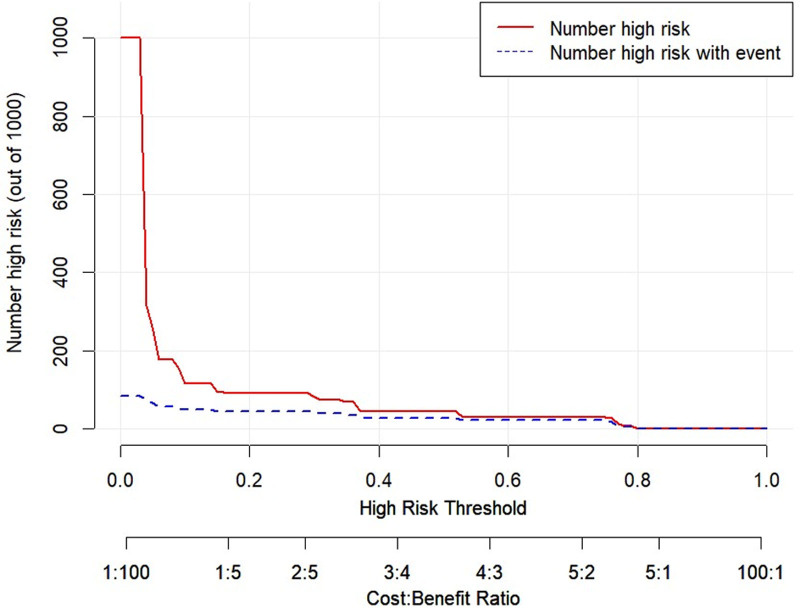
CIC curve for the validation set. In the validation set, threshold probabilities above 40% showed a high consistency between the predicted and actual high-risk cases. CIC = clinical impact curve.

The DCA and CIC were plotted for both the training and validation sets (Figs. 10–13). DCA illustrates the clinical utility of the model across various risk thresholds. The model demonstrates better clinical utility at thresholds where its net benefit surpasses that of “all intervention” or “no intervention” strategies. Results showed that in the training set, the model’s net benefit was significantly higher than “no intervention” and “all intervention” strategies at thresholds between 1% to 68% and 73% to 88%. In the validation set, the model outperformed the other strategies at thresholds of 4% to 72%. The CIC curves depicted the total number of patients predicted to be at high risk and the number of true positives at different risk thresholds. The CIC visually assessed the model’s ability to recognize and accurately predict high-risk patients at specific thresholds, further confirming its clinical effectiveness. In the training set, when the threshold probability exceeded 42%, the high-risk predictions of the model closely matched the actual cases. Similarly, in the validation set, threshold probabilities above 40% showed a high consistency between the predicted and actual high-risk cases. In conclusion, the prediction model demonstrated substantial clinical decision-making benefits in the training and validation sets and has a strong potential for clinical application.

## 4. Discussion

Cholangitis is a common complication after ERCP, with an incidence of approximately 0.5% to 8%, rising above 20% in patients with MBO and stent implantation.^[3,5–7,11,12]^ The condition is also a leading cause of acute postoperative mortality,^[13]^ highlighting the importance of identifying risk factors for clinical management. In this study, we identified diabetes mellitus, previous ERCP, MBO, high bile duct obstruction, and ALB level <35 g/L as independent risk factors for post-ERCP cholangitis through a retrospective analysis. These factors were closely linked to post-ERCP cholangitis and partly aligned with previous literature while offering new insights into the field.

In clinical practice, patients with diabetes mellitus are recognized to have an increased susceptibility to infections. This predisposition is largely attributable to a state of immune dysfunction under hyperglycemia. Key mechanisms include suppressed function of immune cells – such as impaired cytokine production by monocytes and diminished neutrophil activity – as well as dysregulated leukocyte recruitment.^[14–19]^ Concurrent insulin deficiency further exacerbates this milieu by attenuating its inherent anti-inflammatory and immunomodulatory effects.^[20]^ Collectively, these alterations compromise host defense, thereby elevating the risk of post-procedural infections, including cholangitis after ERCP.

A prior ERCP history was identified as an independent risk factor in this study. The procedure itself can induce mechanical injury to the sphincter of Oddi and the bile duct wall, leading to structural alteration and functional incompetence of the sphincter. This may facilitate duodenobiliary reflux and bacterial colonization. Furthermore, patients requiring repeated ERCP often have indwelling biliary stents, which can serve as a nidus for biofilm formation and become a persistent source of infection during subsequent interventions.^[21]^ Although the introduction of exogenous bacteria during a single procedure carries a relatively low immediate risk, the cumulative effect of these mechanisms – sphincter injury, stent colonization, and repeated instrumentation – significantly elevates the infection risk in patients undergoing reoperation.^[22]^

Both MBO and high biliary obstruction were confirmed as significant risk factors. In MBO, tumor invasion not only causes strictures but also induces local inflammation, necrosis, and fibrosis, which disrupt the biliary mucosal barrier and impair bile flow.^[23]^ This is compounded by dysfunction of tight junction proteins and altered bile composition, facilitating bacterial translocation and cholestasis.^[24–27]^ High biliary obstruction, often involving the hepatic hilum, presents with complex anatomy that challenges complete drainage. Even with stenting, suboptimal decompression can lead to persistent biliary stasis – an ideal environment for bacterial proliferation.^[28]^ Moreover, contrast medium retained during the procedure may further irritate the ductal mucosa and worsen drainage^[29]^ Collectively, these pathophysiological alterations account for the markedly higher incidence of cholangitis in such patients, adversely impacting prognosis.

In clinical practice, serum ALB levels <35 g/L or TP levels <60 g/L are defined as hypoproteinemia, which is a significant risk factor for clinical treatment and is closely associated with the prognosis of various diseases and postoperative complications.^[30–32]^ Low albumin levels compromise host defense through multiple interrelated pathways.^[33]^ Albumin plays a critical role in immunomodulation; its deficiency impairs the binding and transport of immune mediators, weakening the systemic inflammatory response. Furthermore, its potent antioxidant and anti-inflammatory properties, including the facilitation of specialized pro-resolving mediators, are diminished, potentially leading to dysregulated inflammation and tissue damage.^[34]^ Clinically, hypoalbuminemia often reflects an underlying state of chronic disease or malnutrition, which is intrinsically linked to immune dysfunction. Finally, albumin is essential for tissue repair and mucosal integrity, meaning its deficiency can delay wound healing and compromise barrier function, thereby facilitating pathogen invasion and impeding recovery.^[35]^

Individualized diagnosis and treatment have become core objectives of modern medicine. Prediction models offer personalized assessments by integrating the specific clinical characteristics of patients and are increasingly utilized in clinical practice.^[36]^ Based on our identified risk factors, we developed and validated a predictive nomogram for post-ERCP cholangitis. The model demonstrated strong discriminative ability and calibration across both training and validation cohorts. DCA confirmed its clinical utility, showing a significant net benefit across a wide range of risk thresholds, while CICs illustrated its accuracy in identifying high-risk individuals. This tool provides a scientific basis for tailored perioperative strategies: aggressive glycemic control and nutritional support for patients with diabetes or hypoalbuminemia, and judicious use of prophylactic antibiotics with ensured biliary drainage for those with prior ERCP, MBO, or high-grade obstruction – an approach supported by existing guidelines for complex biliary cases.^[37]^ In contrast to previous models,^[38,39]^ the predictive model developed in this study was not restricted to specific diseases or procedures, such as choledocholithiasis or biliary stent implantation. Instead, our model comprehensively analyzed all patients who were eligible for ERCP surgery. Although some special cases, such as liver transplant recipients and patients with immune-related biliary diseases, were not included, we thoroughly analyzed the most common cases to construct the model. Another significant advantage of this model is that its key predictors can be easily obtained before surgery. Intuitive and concise line graphs facilitate the clinicians’ understanding and application, thereby enhancing the model’s applicability in real-world clinical settings and laying the foundation for its dissemination.

While this study has several strengths, it also has some limitations. This study was retrospective and based on a limited data sample, which may have introduced selection bias and confounding factors. However, we employed specific exclusion criteria and selection methods to minimize these issues. Additionally, the study included limited imaging data, and the predictive model could be further optimized by incorporating relevant imaging indices. Furthermore, this study focused on an Asian population, which may limit its applicability to other demographic groups. Therefore, large-sample, multi-regional, multi-ethnic, and prospective studies are necessary to further validate and optimize the model.

## 5. Conclusion

In conclusion, the predictive model for post-ERCP cholangitis based on diabetes mellitus, previous ERCP, MBO, high bile duct obstruction, and ALB <35 g/L can assist clinicians in the early identification of high-risk patients, enabling precise interventions and individualized treatment strategies. Optimizing antibiotic regimens, supplementing ALB, and strictly controlling blood glucose levels can effectively reduce the incidence of cholangitis and improve patient prognosis. Additionally, the model provides a new perspective on the development of ERCP-related surgeries, facilitating proactive preventive measures for high-risk patients and enabling timely referrals to higher-level hospitals for comprehensive treatment, thereby maximizing patient safety. For non-high-risk patients, the predictive ability of the model can prevent unnecessary medical interventions, shorten hospitalization duration, reduce costs, and promote day surgery, thereby enhancing medical resource efficiency.

## Author contributions

**Conceptualization:** Wenjie Zhang.

**Data curation:** Wenjie Zhang, Anqi Wei, Guanghua Xie.

**Formal analysis:** Wenjie Zhang, Anqi Wei, Guanghua Xie.

**Methodology:** Wenjie Zhang, Anqi Wei, Guanghua Xie.

**Supervision:** Guanghua Xie.

**Writing – original draft:** Wenjie Zhang, Anqi Wei.

**Writing – review & editing:** Guanghua Xie.
